# P-849. Characteristics and Outcomes of Patients with Community-acquired and Nosocomial *Klebsiella pneumoniae* Bacteremia in a Tertiary Hospital in the Philippines

**DOI:** 10.1093/ofid/ofae631.1041

**Published:** 2025-01-29

**Authors:** Cybele Lara R Abad, Mark Carascal, Justin Allister G Ong

**Affiliations:** University of the Philippines - Philippine General Hospital, Manila, National Capital Region, Philippines; Biomedical Research Unit- Clinical and Translational Research Institute- The Medical City, Manila, National Capital Region, Philippines; The Medical City, Pasig, National Capital Region, Philippines

## Abstract

**Background:**

*Klebsiella pneumoniae* (KPN) bacteremia is associated with higher morbidity and mortality, but there is paucity of local data. This study describes the clinical, microbiologic profile, capsular types, and outcomes of individuals with community-acquired and nosocomial KPN bacteremia.

**Figure:**
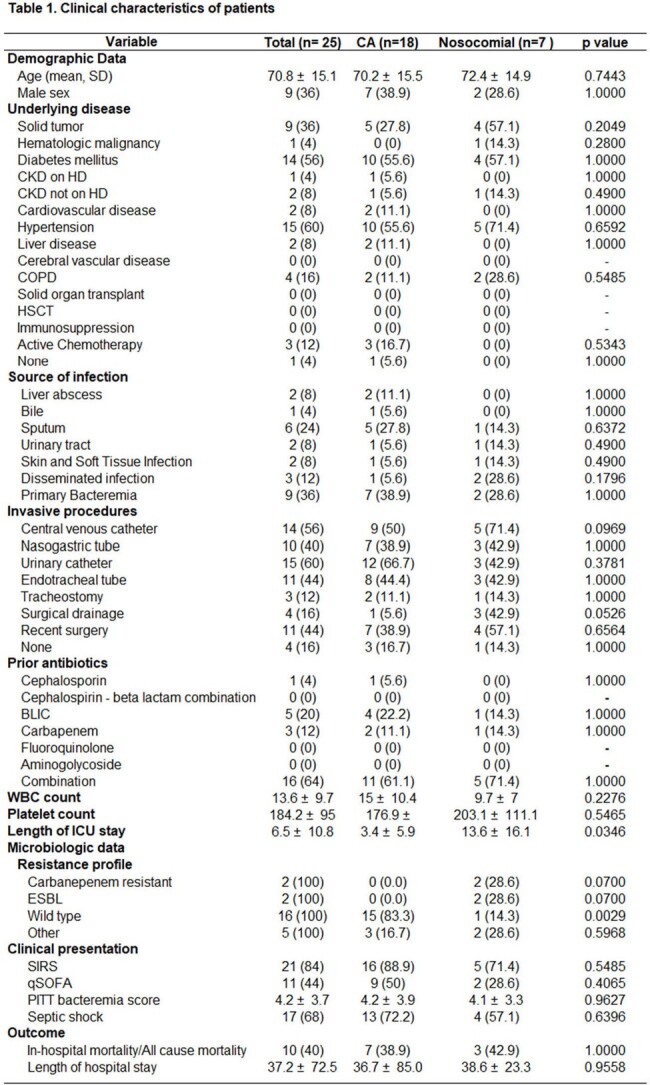

**Methods:**

This study prospectively enrolled patients in a private tertiary hospital from January 1, 2023 to December 31, 2023. Hospitalized patients >/= 18 years old with documented KPN bacteremia were included in the study and isolates subjected to capsular typing. The two-tailed t test was used for continuous variables reported as mean and standard deviation whereas chi-square test was used for categorical variables reported as frequencies and percentages. Descriptive statistics were used and univariate analysis using Medcalc Statistical software.

**Results:**

A total of 25 patients developed KPN bacteremia during the study period; 18 were community-acquired (CA) and 7 were nosocomial. More than half of patients were female (16/25, 64%), with median age 70 (+/- 15) years old. The most common comorbidities were hypertension (15/25, 60%), diabetes mellitus 14/25 (56%), and solid tumor (9/25, 36%). The average intensive care unit stay of 3.4 (+/- 5.9) days for CA KPN bacteremia was significantly shorter than the 13.6 (+/- 16.1) days for those with nosocomial KPN bacteremia. There was no difference in the total length of hospital stay for CA KPN bacteremia (36.7 +/- 85 days) and nosocomial KPN bacteremia (38.6 +/- 23.3 days). Wild-type KPN isolates (15/25, 83.3%) were more commonly found in CA KPN bacteremia, whereas multi-drug resistant isolates were more common for nosocomial KPN. Patients with endotracheal intubation (OR=16, 95% CI 2.2 to 118.3), positive qSOFA (OR=6.42, 95% CI 1.1 to 37.7%), identification of *rmpA* (OR=9.33, 95% CI 1.5 to 59.5%), and K20 capsular serotype (OR=6.0, 95% CI 1.0 to 35.9%) were more likely to have higher mortality.

**Conclusion:**

In this cohort, KPN bacteremia occurred more frequently among women, and in the older age group. CA-KPN was more common than nosocomial KPN bacteremia, but the latter was associated with multi-drug resistance. Endotracheal intubation, positive qSOFA, *rmpA*, and K20 capsular serotypes were associated with higher mortality.

**Disclosures:**

**All Authors**: No reported disclosures

